# In chronic spontaneous urticaria soluble FcεRI is elevated and linked to atopy and chronic inducible urticaria

**DOI:** 10.1002/clt2.12272

**Published:** 2023-07-01

**Authors:** Sherezade Moñino‐Romero, Pavel Kolkhir, Zsolt Szépfalusi, Nicole Schoepke, Martin Metz, Riccardo Asero, Marta Ferrer, Ana Gimenez‐Arnau, Clive E. H. Grattan, Thilo Jakob, George N. Konstantinou, Ulrike Raap, Petra Staubach, Ke Zhang, Carsten Bindslev‐Jensen, Alvaro Daschner, Tamar Kinaciyan, Michael Makris, Nadine Marrouche, Peter Schmid‐Grendelmeier, Gordon Sussman, Elias Toubi, Marcus Maurer, Sabine Altrichter

**Affiliations:** ^1^ Urticaria Center of Reference and Excellence (UCARE) Institute of Allergology Charité – Universitätsmedizin Berlin Corporate Member of Freie Universität Berlin and Humboldt‐Universität zu Berlin Berlin Germany; ^2^ Fraunhofer Institute for Translational Medicine and Pharmacology ITMP, Allergology and Immunology Berlin Germany; ^3^ Division of Pediatric Pulmonology, Allergy and Endocrinology Department of Pediatrics and Adolescent Medicine Comprehensive Center of Pediatrics Medical University of Vienna Vienna Austria; ^4^ Department of Allergology Clinica San Carlo Paderno Dugnano, Milan Italy; ^5^ Department of Allergy and Clinical Immunology Clinica Universidad de Navarra Instituto de Investigación Sanitaria de Navarra (IdiSNA) RETIC de Asma, Reacciones Adversas y Alérgicas (ARADYAL) Pamplona Spain; ^6^ Department of Dermatology Hospital Del Mar IMIM Universitat Autònoma y Universitat Pompeu Fabra Barcelona Spain; ^7^ St John's Institute of Dermatology Guy's Hospital London UK; ^8^ Department of Dermatology and Allergology University Medical Center Giessen and Marburg Justus‐Liebig University Gießen Gießen Germany; ^9^ Department of Allergy and Clinical Immunology 424 General Military Training Hospital Thessaloniki Greece; ^10^ Department of Human Medicine and Health Sciences University Clinic of Dermatology and Allergy University of Oldenburg Oldenburg Germany; ^11^ Department of Dermatology University Medical Center Mainz Mainz Germany; ^12^ Allerdia Inc Los Angeles California USA; ^13^ Department of Dermatology and Allergy Centre Odense University Hospital University of Southern Denmark Odense Denmark; ^14^ Servicio de Alergia Instituto de Investigación Sanitaria (IIS)‐Hospital Universitario de La Princesa Madrid Spain; ^15^ Department of Dermatology Medical University of Vienna Vienna Austria; ^16^ Allergy Unit 2nd Department of Dermatology and Venereology Medical School National and Kapodistrian University of Athens “Attikon” University Hospital Athens Greece; ^17^ Department of Dermatology Norfolk and Norwich University Hospital Norwich UK; ^18^ Allergy Unit Department of Dermatology University Hospital Zürich Switzerland; ^19^ Christine Kühne Center for Allergy Research and Education CK‐CARE Davos Switzerland; ^20^ Division of Allergy and Clinical Immunology University of Toronto Toronto Ontario Canada; ^21^ Faculty of Medicine Bnai‐Zion Medical Center Haifa Israel; ^22^ Department of Dermatology and Venerology Kepler University Hospital Linz Austria

To the Editor,

Chronic Spontaneous Urticaria (CSU) is caused by the activation of skin mast cells (MCs) by various signals including IgG and IgE autoantibodies in autoimmune (type IIb) and autoallergic CSU, respectively.^1^, ^2^ Tests for autoallergic CSU are needed but not available for routine clinical use. Elevated total IgE has been proposed as a biomarker, but study results are inconsistent^3^, ^4^ and total IgE levels in autoallergic CSU may be elevated due to comorbid sensitization rather than the presence of pathogenetically relevant IgE autoantibodies.

Upon IgE‐mediated activation, MCs release the soluble isoform of the high affinity IgE receptor (sFcɛRI), which results in increased serum levels.^5^ Serum sFcɛRI levels have been demonstrated to be elevated and linked to disease activity in patients with IgE‐driven allergies,^6^, ^7^, ^8^ and they are easy to implement in routine clinical practice with commercially available assays. The use of serum sFcεRI in classical allergies has been demonstrated to provide useful information on the clinical relevance of IgE sensitization. Whether or not sFcɛRI levels are elevated in patients with CSU, linked to their total IgE, and associated with clinical features of their disease is currently unknown. To address this, we measured sFcεRI levels retrospectively in the sera of 290 CSU patients and 29 healthy non‐atopic controls (HCs; Table E1 and Online Repository).

Patients with CSU had significantly higher sFcεRI serum levels (median ± IQR: 1.9 ± 62.9 ng/mL) than HCs (1 ± 0.1 ng/mL, *p* < 0.0001; Figure 1A). Half of the CSU patients (47%, 135/290) but only 10% (3/29) of HCs had elevated sFcεRI levels (>2 ng/mL; Table E1). We used the previously established cut‐off for sFcεRI (2 ng/mL) because it defined clinically relevant IgE‐sensitization in an allergic cohort.^6^

**FIGURE 1:**
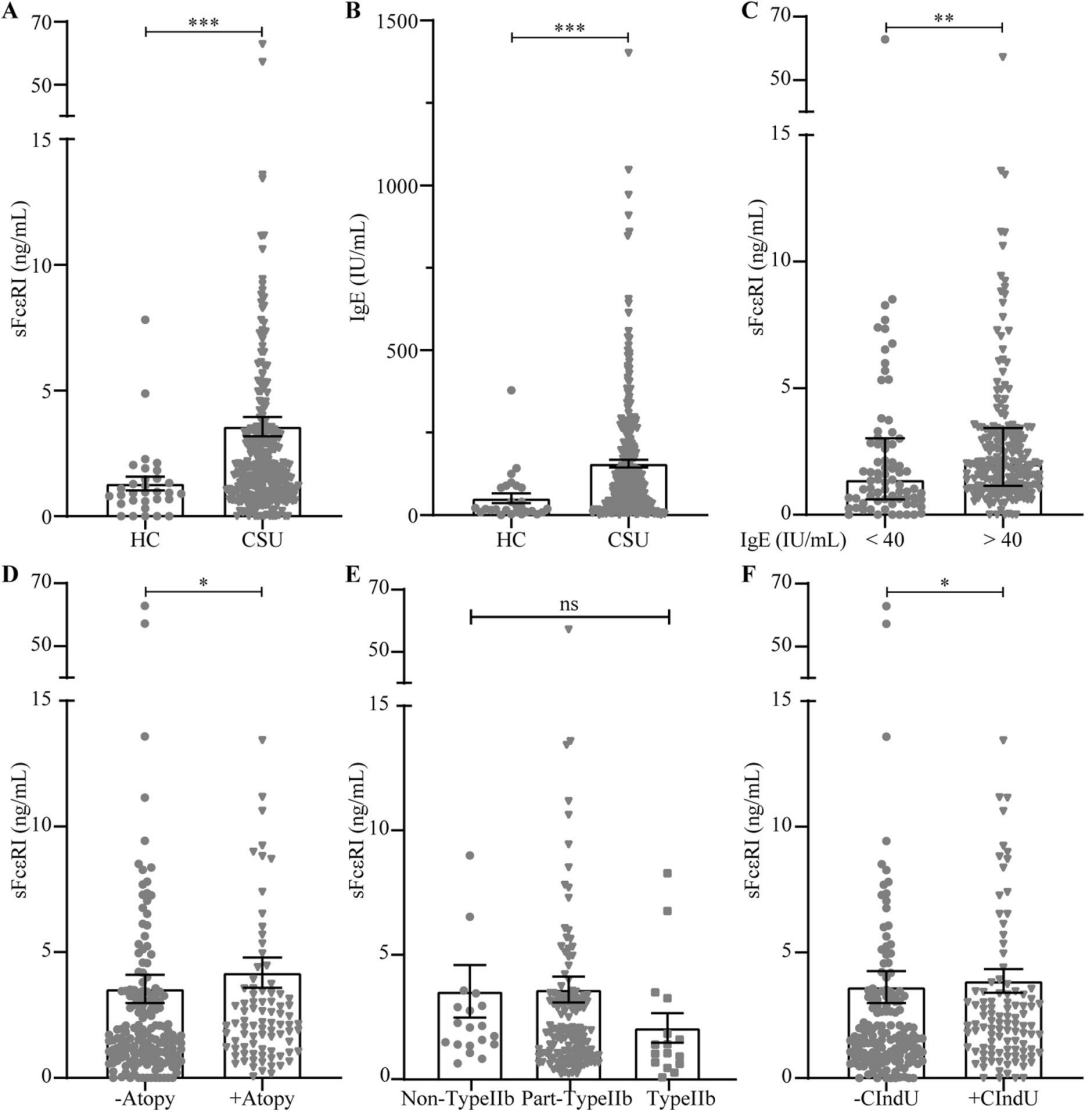
Comparison of serum sFcɛRI (A) and total IgE (B) levels in Chronic Spontaneous Urticaria (CSU) patients and HCs. Serum sFcɛRI levels were compared in CSU patients with low (<40 IU/mL) and normal plus elevated (>40 IU/mL) total IgE levels (C) with and without comorbid atopy (D), endotypes (non‐, part‐ and type IIb, E) or concomitant CIndU (F). Bars represent the median and error bars represent IQR. Mann‐Whitney test was performed; *p < 0.05, **p < 0.01 and ***p < 0.001. CIndU: chronic inducible urticaria; CSU: chronic spontaneous urticaria; HC: healthy controls; IQR: interquartile range.

Total IgE levels (Figure 1B) were also significantly higher in CSU patients (93 ± 1401 IU/mL) than HCs (21 ± 379 IU/mL, *p* < 0.0001) and significantly correlated with sFcεRI levels, albeit weakly (*r* = 0.176, *p* < 0.005). Chronic Spontaneous Urticaria patients with normal and elevated IgE (>40 IU/mL) versus low IgE (<40 IU/mL) had significantly higher sFcεRI levels (*p* < 0.005; Figure 1C). Vice versa, patients with elevated versus normal sFcɛRI levels had more IgE (104 ± 1401 vs. 90 ± 655 IU/mL; *p* = 0.05; Table 1 and Figure E1G). Virtually all serum s was IgE‐bound (*r* = 0.974, *p* < 0.0001; Figure E1A).

**TABLE 1:** Patient characteristics.

	CSU patients with sFcεRI levels		*p* value
	<2 ng/mL (total = 155)	>2 ng/mL (total = 135)	
Age (years; mean, range)	44, 16–76	45, 19–84	0.59^a^
Gender (f:m)	115:39	95:38	0.54^b^
Duration of CSU (months; mean, range)	65, 1–482	65, 2–420	0.95^a^
Atopy (*n*, %)	40/138, 29%	57/126, 45%	0.006^b^
Angioedema (*n*, %)	108/152, 71%	89/131, 68%	0.57^b^
CIndU (*n*, %)	46/137, 34%	62/125, 50%	0.008^b^
Autoimmune CSU (type IIb; *n*, %)	12/97, 12%	4/82, 5%	0.08^b^
UAS7 (mean, range)	18, 0–42	20, 0–42	0.136^a^
Total IgE (IU/mL; mean ± SEM)	128.8 ± 11.04	187.7 ± 20.6	0.05^a^
Total IgE (IU/mL; median ± IQR)	89.95 ± 655	104 ± 1401	

Abbreviations: CIndU, chronic inducible urticaria; CSU, chronic spontaneous urticaria; f, female; IQR, interquartile range; IU, international units; m, male; SEM, standard error of the mean; UAS7, weekly urticaria activity score.

^a^ Mann‐Whitney test.

^b^ Chi‐2 analysis where *p* < 0.05 was considered significant.

sFcɛRI levels in CSU patients were not linked to age or gender, disease duration or activity, angioedema, or ASST (Figure E1 and Table 1), but were significantly higher in those with comorbid atopy (*p* < 0.05) or chronic inducible urticaria (CIndU, *p* < 0.05; Online Repository, Figure 1D,F). Vice versa, rates of comorbid atopy and CIndU were higher in patients with elevated sFcεRI levels (*p* < 0.01; Table 1). There was a trend toward lower sFcɛRI levels in type IIb CSU patients (Figure 1E) defined as triple positivity of autologous serum skin test, basophil tests and presence of IgG autoantibodies by immunoassay (Online Repository). Patients with features of type IIb (triple positive test) or part‐type IIb (at least one positive test) autoimmune CSU were analyzed in the previously reported PURIST study.^9^

Our study, the first on sFcɛRI in CSU, demonstrates that sFcɛRI is elevated in CSU and linked to total IgE and comorbidities. This supports the idea that IgE is a major driver of MC degranulation in CSU, where patients have IgE autoantibodies, for example, IgE to thyroid peroxidase, rather than relevant IgE to allergens. About half of the CSU patients have IgE autoantibodies, similar to the rate of patients with elevated sFcɛRI in our study. Based on our findings, we hypothesize that sFcɛRI may be a suitable marker for autoallergic CSU since it is solely released upon IgE‐mediated crosslinking, that is, in patients with relevant IgE sensitization.

This retrospective analysis has several limitations and further research is ongoing to address the many questions raised by our findings. sFcɛRI levels need to be assessed and compared in CSU patients with and without IgE autoantibodies, and human skin MCs should be investigated for their release of sFcɛRI following activation by IgE versus IgG autoantibodies. Our findings strongly suggest, but do not prove, that sFcɛRI is a biomarker for autoallergic CSU. As such, it may aid individualized treatment in routine clinical practice.

## AUTHOR CONTRIBUTIONS

SMR conceptualization, methodology, formal analysis, investigation, writing – original draft, and visualization. PK methodology and formal analysis. ZS, NS, MMetz, RA, MF, AGA, CEHG, TJ, GNK, UR, PS, KZ, CBJ, AD, TK, MMakris, NM, PSG, GS and ET writing – review and editing and project administration. MMaurer and SA conceptualization, writing – original draft, supervision, project administration and funding acquisition.

## CONFLICT OF INTEREST STATEMENT

SMR, NS, KZ, CBJ, AD, NM and ET have no conflicts of interest. PK was a speaker and/or consultant for Novartis, Roche and ValenzaBio. ZS is or recently was a speaker and/or advisor for Sanofi, Novartis, Nutricia, and AImmune. MMetz has received honoraria as a speaker and/or consultant for Amgen, AstraZeneca, argenx, Celldex, Escient, Jasper Therapeutics, Novartis, Pharvaris, Sanofi‐Aventis, ThirdHarmonicBio. RA is or recently was a speaker and/or advisor for Novartis, ThermoFisher, Sanofi/Genzyme, Menarini, Malesci, GSK. MF has received honoraria (advisory board, speaker) from Novartis, Menarini, Uriach, FAES, Pfizer. MSD and has received a research Grant from GSK and Novartis. AGA or recently was a speaker and/or advisor for and/or has received research funding from Almirall, Amgen, AstraZeneca, Avene, Celldex, Escient Pharmaceuticals, Genentech, GSK, Instituto Carlos III‐ FEDER, Leo Pharma, Menarini, Novartis, Sanofi–Regeneron, Thermo Fisher Scientific, Uriach Pharma/Neucor. CEHG has done consultancy work recently for Celltrion and Sanofi. TJ or recently was a speaker and/or advisor for and/or has received research funding from ALK‐Abello, Allergy Therapeutics/Bencard, Novartis and Thermo‐Fisher Scientific. GNK or recently was a speaker and/or advisor for and/or has received research funding from AstraZeneca, Chiesi, GSK, Menarinin, Novartis, Pfizer, Sanofi, Vianex. UR is or recently was a speaker and/or advisor for Almirall, Abbvie, Janssen, Sanofi, Novartis and UCB. PS is or recently was a speaker and/or advisor for and/or has received research funding from AbbVie, Allergika, Almirall‐Hermal, Amgen, Beiersdorf, Biocryst, BMS, Boehringer‐Ingelheim, Celgene, CSL‐Behring, Eli‐Lilly, Galderma, Hexal, Janssen, Klinge, Klosterfrau, LEO‐Pharma, LETI‐Pharma, L´Oreal, Novartis, Octapharma, Pfizer, Pflüger, Pharming, Regeneron, Shire, Takeda, Regeneron, Sanofi‐Genzyme and UCB Pharma. TK, Tamar Kinaciyan is or recently was a speaker and/or advisor for and/or has received research funding from ALK, Sanofi/Regeneron, Novartis, CSL Behring, Biocryst, Takeda and KalVista. MMakris is or recently was a speaker and/or advisor for and/or has received research funding from Astra Zeneca, Chiesi, GSK, Novartis, Pfizer, Sanofi, Menarini, Elpen, Vianex. PSG or recently was a speaker and/or advisor for and/or has received research funding from AbbVie, Aimmune, ALK‐Abello, Amgen, AstraZeneca, Bencard, Biomed, Bühlmann Diagnostics, Galderma, GlaxoSmithKline, Jansen, LEO, Lilly, L`Oréal, Menarini, Novartis, Pfizer, Pierre Fabre, Roche Pharma, Sanofi Regerenon, Stallergenes and Thermo Fisher. GS is also a medical advisor and/or has received payment for lectures from Novartis, CSL Behring, Pfizer, Abvie, Astra‐Zeneca, Nuvo Pharmaceuticals, and the Allergy Asthma and Immunology Society of Ontario. MMaurer is or recently was a speaker and/or advisor for and/or has received research funding from Allakos, Amgen, Aralez, ArgenX, AstraZeneca, Celldex, Centogene, CSL Behring, FAES, Genentech, GIInnovation, GSK, Innate Pharma, Kyowa Kirin, Leo Pharma, Lilly, Menarini, Moxie, Novartis, Pfizer, Roche, Sanofi/Regeneron, Third Harmonic Bio, UCB, and Uriach. SA or recently was a speaker and/or advisor for and/or has received research funding from AstraZeneca, Allakos, Biocryst, CSL Behring, Sanofi, Takeda, ThermoFisher, Moxie and Novartis.

## FUNDING INFORMATION

Deutsche Forschungsgemeinschaft, Grant/Award Number: RA‐1026/3‐2

## Supporting information

Supporting Information S1

## Data Availability

The data that support the findings of this study are available on request from the corresponding author. The data are not publicly available due to privacy or ethical restrictions.
