# Supplementary material for: In chronic spontaneous urticaria soluble FcεRI is elevated and linked to atopy and chronic inducible urticaria
Source: Clin Transl Allergy. 2023 Jul 1;13(7):e12272. doi: 10.1002/clt2.12272 (PMC10314276; doi:10.1002/clt2.12272)
Supplement: Supplementary file 1 — Supporting Information S1 [file CLT2-13-e12272-s001.docx]

**Online Repository**

**Supplementary Material and Methods**

Patients

A total of 290 patients diagnosed with Chronic Spontaneous Urticaria (CSU) were seen by a specialist at the Department of Dermatology and Allergy, Charité–Universitätsmedizin Berlin or a different Urticaria Center of Reference and Excellence (UCARE). Patients were recruited for the PURIST study at 19 centers (n= 182) or for internal analysis and patient follow up at Charité–Universitätsmedizin Berlin (n= 108). CSU was diagnosed, and its severity was assessed according to the recent EAACI/GA2LEN/EDF/WAO guideline for urticaria^10^. Patient characteristics are given in **Table 1** and **Table E1**, and previously published^9^. Patient blood samples and data were obtained as part of their clinical diagnostic workup, and surplus serum from clinical analyses was stored at –20 for short-term and -80°C for long-term storage until used for this study.

The control group consisted of 29 serum samples from healthy controls (HC) with a negative history of chronic urticaria, autoimmune disease or clinically relevant allergic disease. Clinical characteristics and demographics are presented in **Table E1**.

All patients and controls provided prior informed consent on the use of left-over serum as well as their clinical data for research purposes. All patients’ records were handled in an anonymous manner, following data protection and local ethics (EA1/292/14). As all analyses were performed retrospectively and anonymously, additional ethics approval was not needed or obtained.

Serum samples from CSU patients and HC at Charité were collected in yellow cap BD Vacutainer® SST II Advance tubes to obtain and separate the serum sample. After forming a clot (30 minutes), tubes were centrifuged at 2000 g for 10 minutes at 25˚ C. Next, the serum was stored at -20/80˚ C.

Atopy was defined by IgE sensitization as > 0.35 IU/mL and/or positive skin prick test (SPT) as wheal diameter > 1.5 mm compared to positive control to one or more allergen with or without clinical significance.

Chronic Inducible urticaria (CIndU) was defined as additional urticaria induced by a specific trigger evaluated by a specialist via anamnesis or with according diagnostic tools if available. CIndU included the following: cold, solar, heat, pressure, vibratory, cholinergic and aquagenic urticaria, and symptomatic dermographism.

CSU patients with features of endotypes (type I or type IIb) include the following assays: autologous serum skin test (ASST), basophil activation test (BAT) or basophil histamine release assay (BHRA) and autoantibodies against FcɛRIα as previously described^3^. Limitations in the available data on assays did not allow to perform correlation analysis with sFcεRI levels.

Serum sFcεRI measurement

Samples were diluted, if necessary, in the specific buffer provided with the assay and following manufacturer’s protocol. Serum total sFcεRI levels were analyzed by commercially available ELISA (ThermoFisher Scientific) according to the manufacturer’s protocol. IgE-bound sFcɛRI levels were obtained by detecting serum IgE bound to sFcɛRI as previously described^6,8^.

Statistical analyses

Statistical analyses were performed using Prism 7 (GraphPad Software) or SPSS. Normality test (D’Agostino and Pearson test) failed to confirm normal distribution on sFcεRI and IgE levels from patients, so graphs are shown as median ± IQR of the indicated number of individual data points or independent experiments. Mean ± SEM values for sFcɛRI and IgE are also included in tables. Correlations were calculated by Spearman rank correlation test and correlation coefficients are displayed as “r”. Statistical analysis was performed using Mann-Whitney test for two unmatched groups and Kruskal-Wallis plus Dunn’s multiple correction for multiple comparisons between three or more groups. A p value ≤ 0.05 was considered significant.

**Supplementary figures**


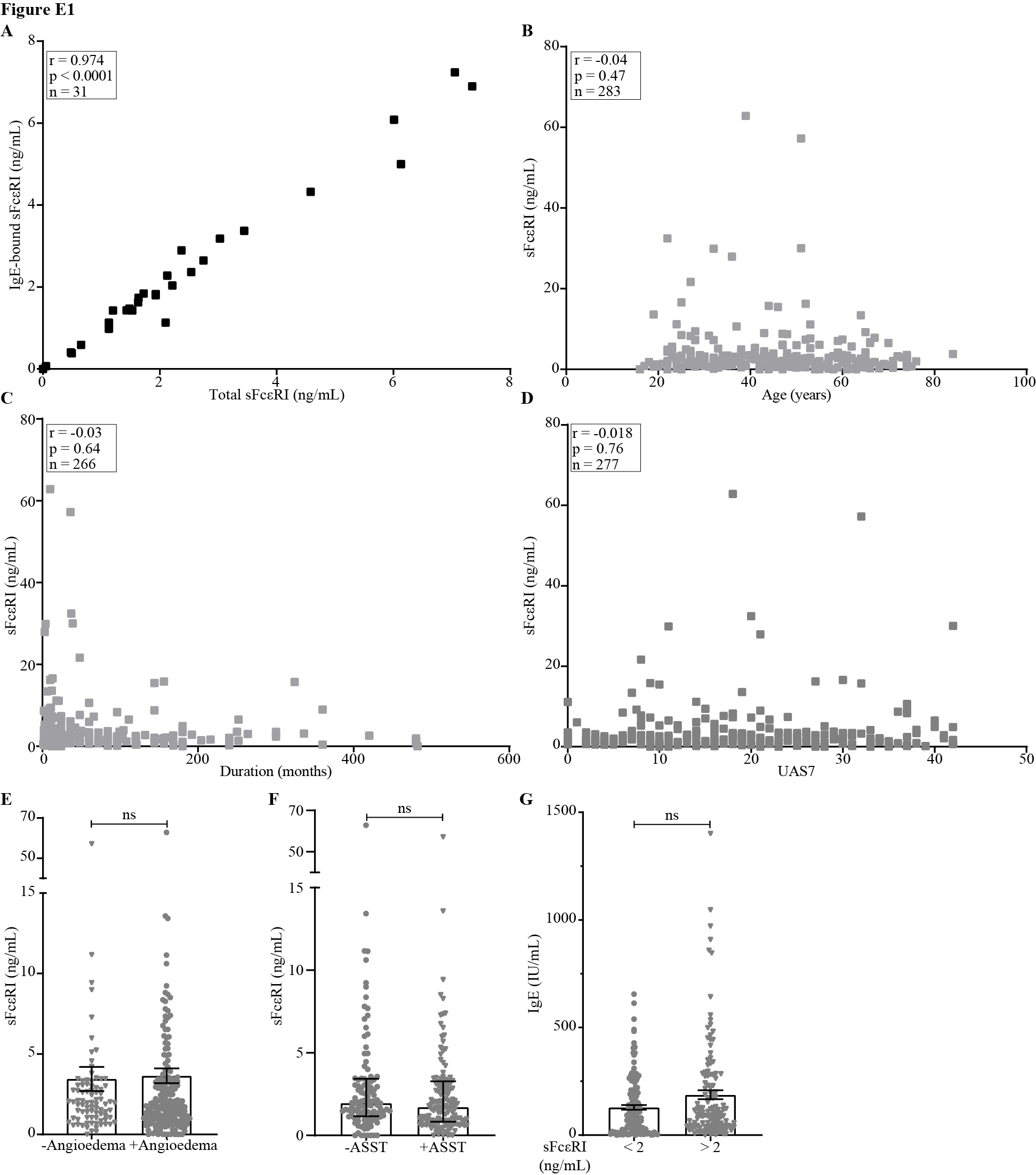


**Figure E1.** Correlation of sFcεRI levels and IgE-bound sFcεRI (**A**), age (**B**), disease duration (**C**), UAS7 (**D**), angioedema (**E**) and ASST (**F**). Total IgE levels in CSU patients with low or elevated sFcεRI levels (**G**). Bars represent the median, and error bars represent IQR. Mann-Whitney test was performed; * = p <0.05. ASST: autologous serum skin test; CIndU: chronic inducible urticaria; CSU: chronic spontaneous urticaria; IQR: interquartile range; ns: not significant; UAS7: weekly urticaria activity score.

**Supplementary tables**

**Table E1**

|  | **CSU (n = 290)** | **HC (n = 29)** | **p value** |
| --- | --- | --- | --- |
| **Age (mean, range)** | 45, 16-84 | 39, 19-64 | 0.058^†^ |
| **Gender (f:m)** | 210:76 | 19:10 | 0.38^‡^ |
| **Duration of CSU (months; mean, range)** | 65, 1-482 | – | – |
| **Atopy (n, %)** | 97/264, 37% | 0/31, 0% | **< 0.0001**^‡^ |
| **Angioedema (n, %)** | 197/283, 70% | 0/31, 0% | **< 0.0001**^‡^ |
| **CIndU (n, %)** | 107/262, 41% | 0/31, 0% | **< 0.0001**^‡^ |
| **Autoimmune CSU (type IIb; n, %)** | 16/179, 9% | 0/31, 0% | 0.1^‡^ |
| **sFcεRI > 2 ng/ml (n, %)** | 135/290, 47% | 3/29, 10% | **0.0002**^‡^ |
| **sFcεRI (ng/mL, mean ± SEM)** | 3.57 ± 0.38 | 0.96 **±** 0.12 | **< 0.0001**^†^ |
| **sFcεRI (ng/mL, median ± IQR)** | 1.87 ± 62.86 | 0.92 **±** 2.23 |  |
| **IgE (IU/mL, mean ± SEM)** | 156.42 ± 11.40 | 53.81 ± 15.95 | **< 0.0001**^†^ |
| **IgE (IU/mL, median ± IQR)** | 93 ± 1401 | 21.2 ± 378.8 |  |
| **IgE low (< 40 IU/mL; n, %)** | 77/275, 28% | 18/25, 72% | **< 0.0001**^‡^ |
| **IgE normal and elevated (> 40 IU/mL; n, %)** | 198/275, 72% | 7/25, 28% |  |
| **IgE elevated (> 100 IU/mL; n, %)** | 132/275, 48% | 3/25, 12% | 0.0005^‡^ |

CIndU: chronic inducible urticaria; CSU: crhonic spontaneous urticaria; f: female; IQR: interquartile range; IU: international units; m:male; SEM: standard error of the mean. ^†^Mann-Whitney test or ^‡^Chi-2 analysis was performed where p < 0.05 was considered significant and is represented in bold.

**Online repository references**

Zuberbier, T., Abdul Latiff, A. H., Abuzakouk, M., Aquilina, S., Asero, R., Baker, D., . . . Maurer, M. (2022). The international EAACI/GA²LEN/EuroGuiDerm/APAAACI guideline for the definition, classification, diagnosis, and management of urticaria. *Allergy*, *77*(3), 734-766. <https://doi.org/10.1111/all.15090>
